# Unravel the Supremacy of *Klebsiella variicola* over Native Microbial Strains for Aroma-Enhancing Compound Production in Reconstituted Tobacco Concentrate through Metagenomic Analysis

**DOI:** 10.3390/metabo14030158

**Published:** 2024-03-08

**Authors:** Shen Huang, Li Zhu, Ke Wang, Xinlong Zhang, Duobin Mao, Aamir Rasool

**Affiliations:** 1 College of Tobacco Science and Engineering, Zhengzhou University of Light Industry, Zhengzhou 450002, China; huangshen@zzuli.edu.cn (S.H.); ezhou115@gmail.com (L.Z.); jiak_w@163.com (K.W.); zxlzxl0127@163.com (X.Z.); 2 Institute of Biochemistry, University of Balochistan, Quetta 87300, Pakistan

**Keywords:** microbial community, metagenome, *Klebsiella variicola*, reconstituted tobacco leaf concentrate, neutral flavor components, sensory attributes

## Abstract

Sensory attributes strongly influence consumers’ preferences for products. The inoculation of the *Klebsiella variicola* H8 strain in a reconstituted tobacco leaf concentrate (RTLC) solution increased neutral aroma-enhancing compound (NAEC) production by 45%, decreased the nicotine level by 25%, decreased the water-soluble total sugar content by ~36%, and improved the sensory quality by 5.71%. The production of NAECs such as dihydrokiwi lactone (DHKL: 192.86%), 1,2,3,4-tetrahydro-1,1,6-trimethylnaphthalene (THTMN: 177.77%), 2,4-di-tert-butylphenol (DTBP: 25%), 4-oxoisofolkone (OIFK: 116.66%,) 1,9-heptadecadiene-4,6-diyn-3-ol (HDD: 116.67%), β-damastrone (BDS: 116.67), and megastigmatrienone A (MSTA: 116.67%) was increased. A metagenomics analysis of the microbial community in the fermented RTLC (FRTLC) was performed to elucidate the mechanism by which NAECs were produced. As a result, 24 groups of functional genes were identified, and among them, five families of carbohydrate-active enzymes, (i) glycoside hydrolase (GH), (ii) glycosyltransferase (GT), (iii) polysaccharide lyase (PL), (iv) carbohydrate esterase (CE), and (v) auxiliary active enzyme (AA), were found to be positively correlated with the production of NAECs. However, among the GHs, the GHs annotated from the H8 strain chromosome displayed the highest relative abundance and a positive correlation with the production of NAECs. Specifically, the GH13-14, GH13-20, GH13-38, GH13-25, GH13-10, GH42, and GH28 genes of the H8 strain were relatively more abundant and were key contributors to the production of NAECs. The correlation analyses revealed that the H8 strain plays a leading role among all the microorganisms in FRTLC in the production of NAECs. Our findings support the application of *Klebsiella variicola* in NAEC production and a reduction in nicotine content in tobacco products.

## 1. Introduction

Tobacco (*Nicotiana tabacum* L.) is a high value crop. A plethora of efforts are focusing on enhancing the neutral aroma-enhancing compound (NAEC) content and reducing the nicotine level in reconstituted tobacco leaf concentrates (RTLCs), because their sensory attributes strongly effect their economic value. At present, various strategies, including variety improvement, improvement of cultivation measures, improvement of tobacco leaf mixing technology, and the addition of microbial spices are being used to enhance the production of NAECs and reduce the amount of harmful compounds. High-end microbial engineering technologies have enabled researchers to customize the production of NAECs and nicotine in RTLCs by using microorganisms [1,2,3]. The employment of microorganisms in the fermentation of RTLCs has been shown to have many benefits including effectively increasing the color, reducing the pungent smell, and eliminating the bitter taste [1,2]; reducing the content of tar and other harmful substances [3,4]; and improving its combustibility and decreasing air pollution [5,6,7].

The reconstituted tobacco leaf concentrates are manufactured from tobacco stems and tobacco powder through a papermaking process [8,9]; during this process, essential aroma substances in the tobacco are lost [4]. Therefore, the production of aroma compounds in tobacco can be increased through employing microorganisms in the fermentation of RTLCs [10,11]. The inoculation of *Acinetobacter* on cigar tobacco leaves (CTLs) significantly enhanced the sensory quality of its fermented products [12]. The complementary culture of bacteria (*Erwinia carotovora*) and fungi (*T. longibrachiatum*) effectively degraded pectin and cellulose by producing pectin- and cellulose-degrading enzymes, which could then be used for the production of NAECs [13].

Microorganisms produce a range of enzymes that can transform harmful compounds into aromatic compounds and enhance the sensory attributes of tobacco [14,15]. The *Klebsiella* genus produces an important enzyme (β-galactosidase), which hydrolyzes the β-galactosidic bonds present in oligosaccharides and polysaccharides [16,17,18,19]. *Klebsiella* sp. 5246 also expresses pullulanase that degrades branched α-glucans [20]. The *Klebsiella aerogenes* type 25 expresses an extracellular alginolyase that specifically hydrolyzes the α-l-guluronosyl linkages in whole alginate [21]. The simple sugar molecules released from these reactions act as the precursors for the production of NAECs [22].

In this study, the *Klebsiella variicola* H8 strain was tested for its ability to enhance the production of NAECs and reduce the nicotine level in a fermented RTLC (FRTLC). A metagenomic sequencing analysis was performed to determine the contribution of the *Klebsiella variicola* H8 strain in the production of NAECs combined with the native microbial community in the RTLC. Additionally, various statistical correlation analyses were performed to determine the relationship between *Klebsiella variicola* H8, native microorganisms, and the enzymes produced by these microorganisms in the conversion of the nutrients present in the RTLC into NAECs. The *Klebsiella variicola* is an opportunistic pathogenic bacterium that mainly colonizes plants and is also a normal colonizing bacterium in the human body [23,24]. Under normal circumstances, it will not cause disease. Huang Hong and others [25] conducted research on *Klebsiella variicola*, but no virulence genes were detected. 

In this study, we primarily tested the utilization of microbial strains like *Klebsiella variicola* H8 to customize aroma profiles and reduce the nicotine content. Consequently, the study will contribute to the manufacturing of tobacco products with enhanced sensory attributes and potentially reduce the health risks caused by nicotine. However, we will conduct proper research on the safety of utilizing *Klebsiella variicola* H8 in tobacco in the prospective study.

## 2. Materials and Methods

### 2.1. Experiment Materials, Strain, Fermentation Media and Conditions

The RTLC (brand name: TS-006) was gifted by China Tobacco Henan Industrial Co., Ltd. (Xuchang, China). The important features of the reconstituted tobacco concentrate are as follows: dry mass, 40%; Baume degree, 26 °Bé; density, 1.22 g/mL; and pH 6. 

The *Klebsiella variicola* H8 strain was chosen for this study because, during the screening process, we found that it outperformed strains of *Pantoea* H4, *Acinetobacter* H9, *Staphylococcus* H11, and *Enterobacter* H12 in producing NAECs. Additionally, we took into account the scores awarded by cigarette evaluation experts, as well as the metabolic profile of microorganisms and their heat production efficiency. These factors indicated that the H8 strain is the most suitable and effective bacterium for enhancing NAECs. We employed two different temperatures because *Klebsiella variicola* H8 grows better at 37 °C, while at 30 °C it produces a higher amount of NAECs.

An LB culture medium was used for the cultivation of the *Klebsiella variicola* H8 strain and the medium contained the following components: NaCl (10 g/L), tryptone (10 g/L), and yeast extract (5 g/L). A PBS solution was prepared by dissolving 8.0 g NaCl, 0.2 g KCl, 1.44 g Na_2_HPO_4_, and 0.24 g KH_2_PO_4_ in 800 mL of distilled water. The pH was adjusted using HCl, and the final volume adjusted to 1 L using distilled water.

The *Klebsiella variicola* H8 strain was activated by growing it in LB broth at 37 °C for 12 h with rotary shaking at 150 rpm. Subsequently, the cells of *Klebsiella variicola* H8 were isolated from the broth through centrifugation at 12,000 rpm for 12 min at 4 °C. The cell pellet was then thrice washed with PBS. Afterward, these pellets were re-dissolved and inoculated into a fermentation tank containing 3 L of RTLC at a 7% (*v*/*v*) ratio. The fermentation of the RTLC was carried out at 37 °C with a stirring speed of 150 rpm, and the inoculum size was around 6.3 × 10^7^ CFU/g (7%). The samples for analysis were obtained from the fermentation tank at the following time intervals in triplicate and tagged accordingly: 0 h (CK), 8 h (H8H), 16 h (H16H), 24 h (H24H), and 36 h (H36H). The tobacco concentrate fermented for 0 h was used as the control (blank) throughout this study for comparisons of the data from the metagenomic analysis, GC-MS analysis, production of routine chemical components during the fermentation of the RTLC by *Klebsiella variicola* H8, and evaluation of the quality of cigarettes made from the FRTLC. The RTLC was not sterilized to mimic the industrial tobacco fermentation milieu. All chemical reagents are sourced from Shenggong Biotechnology Co., Ltd. (Shanghai, China).

### 2.2. Whole-Genome Shotgun Sequencing for Metagenomic Sequence Analysis

The whole-genome shotgun sequencing metagenomic sequence analysis of each sample was conducted using the Illumina NovaSeq/HiSeq High-Throughput Sequencing platform (Illumina, San Diego, CA, USA) at Shanghai Paralon Biotechnology Co., Ltd., (Shanghai, China). Genomic DNA was extracted from the microbial community in the FRTLC solution. The extracted DNA was then randomly fragmented into small fragments, and libraries of these fragments were constructed for pair-end sequencing. This process resulted in pair-end sequenced libraries of DNA fragments, allowing for the metagenomic sequencing of the microbial community in each sample.

The quality of the sequence data generated by the Illumina sequencing platform was assessed using the FastQC quality control tool (https://www.bioinformatics.babraham.ac.uk/projects/fastqc/, accessed on 15 October 2022), and the Cutadapt bioinformatics tool (https://cutadapt.readthedocs.io/en/stable/, accessed on 15 October 2022), was employed to remove adapter sequences, primers, and other unwanted or low-quality portions of the sequenced data. We obtained high-quality sequence data through the use of these tools. The concentration of the metagenome samples was assessed using a NanoDrop™ 2000/2000c Spectrophotometer (Thermo, Waltham, MA, USA) and a concentration of 10 ng/μL was used for the NGS sequencing.

The sequenced data were used in the following analyses: functional analysis, taxonomic profiling, diversity analysis, community structure analysis, pathway analysis, functional annotation, and metagenomic assembly. 

### 2.3. Gene Prediction

The sequence data obtained from whole-genome shotgun sequencing were analyzed using GeneMarkS version 4.28. This software precisely predicts the genes and their locations in the sequenced genome. GeneMarkS is specifically designed for the prediction of genes in prokaryotes, intron-less eukaryotes, eukaryotic viruses, phages, and EST/cDNA sequences (http://exon.gatech.edu/genemark/genemarks.cgi, accessed on 15 October 2022).

### 2.4. GC-MS Analysis of FRTLC

The production of NAECs and the degradation of nicotine during fermentation by the *Klebsiella variicola* H8 strain and native microbial strains was determined using GC-MS (Agilent, Santa Clara, CA, USA) [26,27] and was quantified in all five samples, CK (blank), H8 (8 h), H16 (16 h), H24 (24 h), and H36 (36 h), each in triplicate. For the extraction process, 20.5 mL of the sample was added to a 1000 mL round-bottom flask containing ddH_2_O (500 mL), 2,6-dichlorotoluene (100 μL), and dichloromethane (100 mL). The mixture was then heated at 60 °C for 2.5 h under atmospheric pressure. The resulting extract was concentrated to 1 mL for the analysis of NAEC and nicotine content. 2,6-dichlorotoluene (98.0%) was used as the extraction solvent and internal standard for the GC-MS analysis.

The GC-MS system consisted of an Agilent 190918-436 (Agilent, Santa Clara, CA, USA) and an Agilent 5975 mass spectrometer (Agilent, Santa Clara, CA, USA), and the GC device had a fused capillary column coated with HP5-MS (60 m × 0.25 mm × 0.25 um). The auto-sampler set at 1 mL was used for sample injection, and the injection port temperature was set at 280 °C. Helium (99.999%) was used as the carrier gas, and the flow rate was set at 1.0 mL/min. The initial column oven temperature was set at 50 °C, maintained for 4 min, and then increased to 240 °C at a rate of 2 °C/min, which was maintained for 5 min. Approximately 99% pure standards of neutral aroma components and nicotine were used for quantification purposes.

Finally, neutral aroma components and nicotine were identified by comparing the obtained mass spectra with the NIST library database, published data, and reference compounds [26,28].

### 2.5. Determination of Routine Chemical Components 

In this study, the routine chemical components of all five samples (CK, H8, H16, H24, and H36), each in triplicate, were analyzed. The key routine chemical components (total sugar, reducing sugar, total alkaloid, and potassium) were assessed using the continuous flow methods specified by the tobacco industry standards, such as YC/T 159-2019 [S], YC/T 161-2002 [S], YC/T 162-2011 [S], and YC/T 468-2013 [S]. Three sets of controls corresponding to each time interval were established by growing the RTLC without the addition of the *Klebsiella variicola* H8 strain.

### 2.6. Evaluation of the Quality of Cigarettes 

Tobacco cigarettes were manufactured from the FRTLC obtained from all samples and control, in triplicate. The assessment of cigarette quality was conducted by a team of 9 staff members from Henan Cigarette Industry Tobacco Thin Sheet Co., Ltd., (Xuchang, China) each with 10 years of experience in testing cigarette quality.

In the evaluation process, cigarettes made with the 0-hour fermentation were first tested and scored. Subsequently, cigarettes made with the sample tobacco concentrates (8-h,16-h, 24-h and 36-h) were tested and scored. The evaluation criteria were based on the following indicators: concentration of smoke, aroma quality, aroma quantity, woody odor, aftertaste, unpleasant odor, and irritation. We used the score of the cigarettes made blank as the benchmark, and added or subtracted 0.1 points from this baseline score, and the data collected from the 9 evaluators were combined for each cigarette, with the average of the individual scores recorded as the final score.

## 3. Results

### 3.1. Dynamics of Microbial Community and Functional Genes in FRTLC

#### 3.1.1. Microbial Interactions and Fermentation Dynamics 

The base pairs are vital indices for assessing the complexity and size of the metagenomic data generated during the sequencing process. The results of the metagenome sequencing revealed significant variations in the total number of base pairs at different time intervals of fermentation, namely 0 h (6,661,518,900 bp), 8 h (6,123,673,200 bp), 16 h (639,737,600 bp), 24 h (5,835,658,000 bp), and 36 h (6,518,654,300 bp). Notably, the total number of base pairs exhibited different changes over time, possibly due to changes in the microbial community structure, which commonly take place during the fermentation process.

The N50 is a metric indicating the contiguity of an assembly, and a higher N50 value indicates a better continuity in the assembled sequences. In our study, the N50 value for each sample group surpassed 500 bp, and the assembly featured a maximum contig length of 4640 bp, collectively indicating a high-quality sequence assembly. This assembly was subsequently used as a reliable reference sample for the subsequent analyses of the next-generation sequencing results.

From the metagenomic sequencing data, 168 distinct microbial species were identified. Interestingly, the RTLC fermented for 8 h displayed 63 unique microbial species. However, no additional unique microbial species were detected in the RTLCs fermented for 16, 24, and 36 h (Supplementary Figure S1). 

This finding suggests that during the first 8 h of fermentation, the microbial community expanded due to the abundance of nutrients. However, during the later stages of fermentation, the microbial community reached an equilibrium. This highlights the significance of time intervals in controlling the diversity of the microbial community.

**Figure 1:**
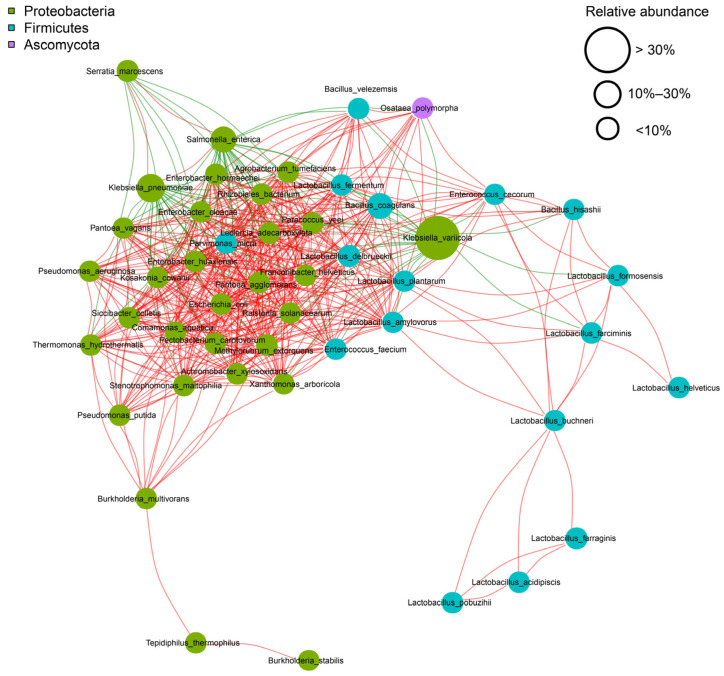
Correlation diagram of dominant microorganisms. Each node represents a dominant bacterium. The size of each node corresponds to the average relative abundance of the corresponding species. Connections between nodes indicate the correlation between two species. More connections indicate a stronger correlation. Red lines represent positive correlations, while green lines represent negative correlations.

The metagenomic sequencing data demonstrated that bacterial species were relatively more abundant in the FRTLC compared to fungal species. The bacterial species of the phyla *Proteobacteria* and *Firmicutes* were the predominant bacterial species in the FRTLC (Figure 1).

**Figure 2:**
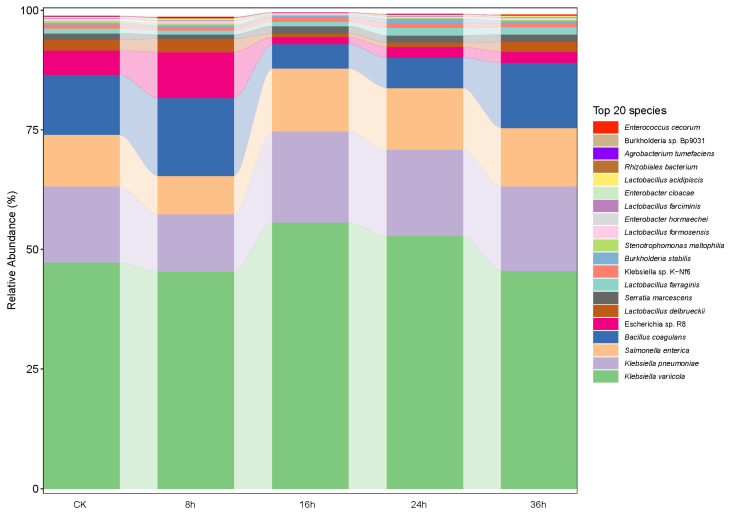
Histogram of relative abundance of dominant strains. The length of each bar on the ordinate represents the relative abundance of the corresponding taxon in the sample. The longer the bar, the higher the relative abundance of the taxon in that sample.

Among the *Proteobacterial* species, *Klebsiella variicola* was found to be a relatively more dominant species compared to (in descending order of their abundance in the FRTLC) *Klebsiella pneumoniae*, *Salmonella enterica*, *Bacillus coagulans*, *Escherichia* sp. *R8*, *Lactobacillus delbrueck*ii, and *Serratia* (Figure 2). The metagenomic sequencing data demonstrated that during the fermentation of RTLC, the abundance of *Klebsiella variicola*, *Klebsiella pneumoniae*, *Serratia*, and *Salmonella* initially increased and reached its peak after 16 h, after which it decreased as the fermentation process progressed (24 and 36 h) (Figure 2). The data also showed that the relative abundance of *Lactobacillus*, *Lactobacillus delbrueckii*, *Escherichia* sp. *R8*, *Burkholderia*, and *Bacillus coagulans* decreased as the fermentation of the RTLC progressed. Furthermore, the relative abundance of other microorganisms, such as *Enterococcus* and *Pantoea*, also decreased during the fermentation process (Figure 2). 

The relative decrease in the abundance of microorganisms in the FRTLC during the fermentation process is likely due to the competitive inhibition of *Klebsiella variicola*. As the major strain in the fermentation ecosystem, *Klebsiella variicola* may have outnumbered and outcompeted the other microorganisms for essential resources, leading to a decline in their population. Additionally, the decrease in the relative abundance of the other microorganisms could also have resulted from a combination of various ecological factors that influenced the microbial community structure of the FRTLC [29,30]. 

**Figure 3:**
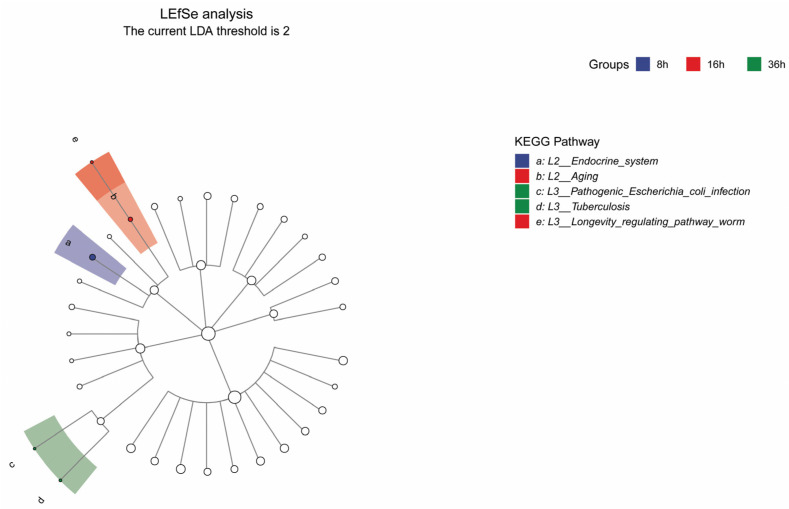
Branch diagram of LDA value distribution of different functional groups. This diagram illustrates the topological relationship of the main functional groups in the sample community from KEGG path level 1 to level 3 (from the inner circle to the outer circle). Node size corresponds to the average relative abundance of the functional group. Colored nodes indicate taxa that exhibit significant inter-group differences, with higher abundance in the group samples represented by the color. Hollow nodes represent no significant differences between groups.

The metagenomic sequencing data served as the foundation for determining gene abundance. The differential analysis of microbial genes indicated an increase in the relative abundance of endocrine system-related genes in the RTLC fermented for 8 h (Figure 3). Endocrine system-related genes regulate nutrient metabolism, impact signaling pathways, affect host interactions, influence the metabolism of secondary metabolites, and induce pH and environmental changes by altering the physiology of the host [31,32]. After 16 h of fermentation, the shift in the gene abundance led to changes in the relative abundance of the microorganisms. The decrease in microbial abundance was attributed to reduced nutrient availability, coupled with an increase in enzymes of genes related to aging and lifespan regulation pathways (K08339, K06067, K12762, K12767, and K01110) in the microbial community of the FRTLC (Figure 3). The increased abundance of the key enzyme ATG5, which is associated with cell aging and death, further contributed to the decline in microbial abundance. Following this, pathogenic microorganisms began to proliferate after 36 h of fermentation, leading to an increase in the relative abundance of their pathogenic genes (Figure 3). These changes were closely associated with the microorganisms in the concentrate, particularly *Klebsiella variicola* (Supplementary Figure S2). 

#### 3.1.2. Analysis of FRTLC Microbial Gene Function 

The databases GOSlim, KEGG, eggNOG, and CAZy are widely used in microbial gene function analyses since they offer a concise categorization of gene functions, a comprehensive platform for pathway and functional information, an analysis of gene functions based on evolutionary relationships, and information on enzymes related to carbohydrate metabolism, respectively. 

**Figure 4:**
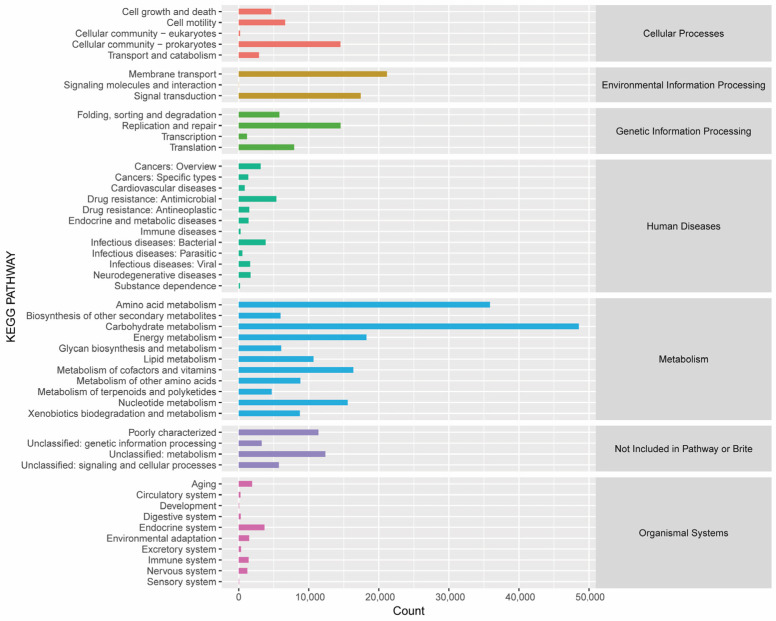
Statistical chart of KEGG metabolic pathway annotation results. The time points indicated on the KEGG metabolic pathway map represent the average values across five specific time points: 0 h, 8 h, 16 h, 24 h, and 36 h.

The high-quality sequencing data obtained from whole-genome sequencing were compared with these databases, and the annotation results revealed that the majority of functional genes crucial for the fermentation of the RTLC originated from bacteria, with only a few originating from fungi. Most of the genes expressed by the microorganisms involved in the fermentation of the RTLC are involved in pathways such as metabolism, genetic information processing, signal transduction, cellular processes, and cellular environmental information processing pathways. The genes involved in metabolic processes, such as amino acid metabolism and carbohydrate metabolism, constituted a relatively large proportion of the other genes. Additionally, the genes related to environmental information processing, such as membrane transport and signal transduction, represented the second-largest proportion (Figure 4). The higher relative abundance of genes involved in metabolic processes and environmental information processing highlights the active participation of microorganisms in the fermentation of the metabolites in the RTLC and their responsive behavior to changes in their surroundings during the fermentation process.

**Figure 5:**
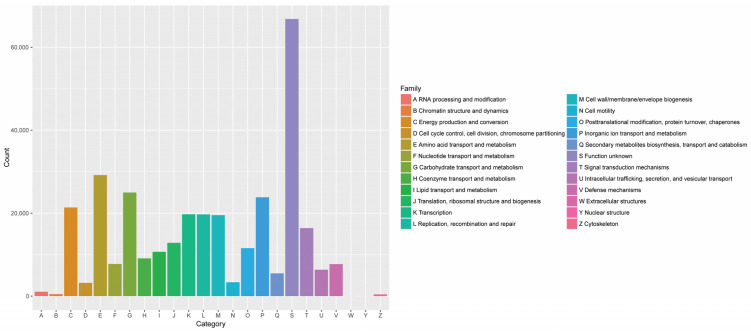
Statistical chart represents groups of functional genes annotated with the eggNOG database. The abscissa corresponds to 24 groups of functional genes, whereas the ordinate displays the number of annotated functional gene groups.

The statistical chart of the eggNOG annotations quantitatively represents the 24 groups of functional genes that were identified through whole-genome sequencing of the microorganisms involved in the fermentation of the RTLC. These results showed that there was a higher relative abundance of genes related to the amino acid, lipid, carbohydrate, and polyketone metabolism pathways, indicating that they have a significant role in the fermentation of RTLCs (Figure 5). These metabolic pathways play a critical role in the production of NAECs by converting amino acids, lipids, and carbohydrates into secondary metabolites [33,34,35]. It is speculated that aroma-enhancing bacteria initially adapt to the extreme conditions of the RTLC and subsequently secrete extracellular enzymes into the medium to convert macromolecules and other substances into NAECs [36].

**Figure 6:**
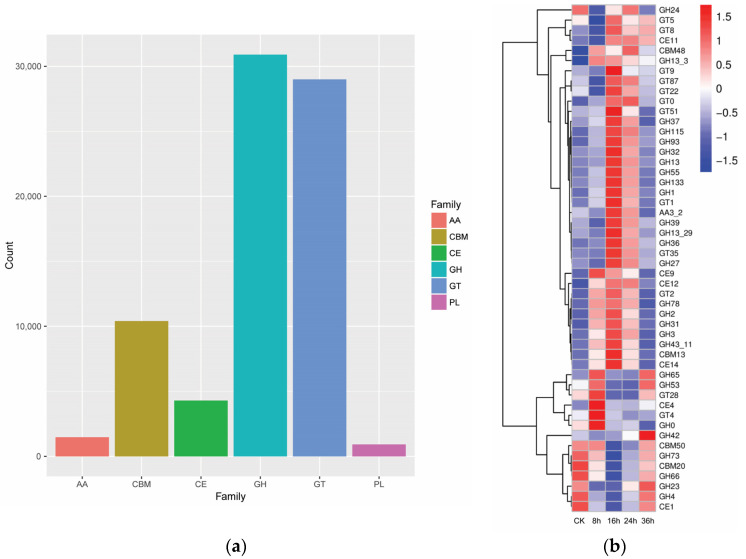
(**a**) Statistical chart of CAZy annotations. The abscissa presents the CAZy families, and the ordinate presents the number of members in each CAZy family, which include the GH, GT, PL, CE, and AA families. (**b**) Statistical heat map of the relative abundance of carbohydrate-active enzymes.

The statistical chart of the CAZy annotations presents the relative abundance of different families of carbohydrate-active enzymes from the microorganisms participating in the fermentation of the RTLC. As a result, five families of carbohydrate-active enzymes, namely (i) glycoside hydrolase (GH), (ii) glycosyltransferase (GT), (iii) polysaccharide lyase (PL), (iv) carbohydrate esterase (CE), and (v) auxiliary active enzyme (AA) were identified from the metagenomic data (Figure 6a). The relative abundance of these families was recorded as 3,172,429,819, 9,344,397, and 1515 counts, respectively. The GH genes exhibited the highest relative abundance compared to the other carbohydrate-active enzymes identified from the metagenomic data. A further analysis of the metagenomic data also revealed an increase in the relative abundance of 19 GH and 9 GT genes at the 16th and 24th h of fermentation, respectively. The relative abundance of the GH24, GH13, GH13-3, GH37, GH32, GH1, GH39, GH13-29, GH36, GH78, GH2, GH31, and GH3 genes in the whole-genome sequence data of samples fermented up to 16 h was significantly increased (Figure 6b and Supplementary Table S2). 

The routine chemical components, such as the total sugar, reducing sugar, total alkaloid, and potassium levels, serve as basic indicators for assessing the quality of tobacco in formulation design [37], quality monitoring [38], and classification of cigarette products [39]. In conjunction with the above findings, the results of the GC-MS analysis of the routine chemical components of the FRTLC were consistent with the data of the statistical chart of CAZy and demonstrate that the water-soluble total sugar content consistently decreased throughout the fermentation process (Supplementary Table S1). However, the highest decline in the water-soluble total sugar content, approximately ~19.106%, was observed between 8 and 16 h of fermentation. The total alkaloid content initially decreased by ~5.22% up to the 16th h of fermentation but it later increased by ~4.72. The water-soluble total sugar content decreased by 36.01% and the nicotine level decreased by ~25% in the FRTLC after 36 h of fermentation. 

These findings align with the notion that carbohydrate-active enzymes perform the biochemical conversion of the chemicals present in the FRTLC to produce NAECs (Table 1 and Supplementary Table S1).

### 3.2. Correlation between Microbial Growth, NAECs, and Nicotine Level in FRTLC

The GC-MS analysis of the FRTLC revealed that the conversion of metabolites into NAECs by microorganisms reached the highest level after 24 h of fermentation and was at its lowest level after 36 h (Table 1). These NAECs included benzyl alcohol, phenylethanol, 2-acetyl-pyrrole, linalool, MSTA, megastigmatrienone b, megastigmatrienone c, megastigmatrienone d, β-damasone, solanone, and damasone. The production of dihydrokiwi lactone (DHKL) significantly increased (192.86%) compared to the other NAECs. The production of other aromatic compounds, like 1,2,3,4-tetrahydro-1,1,6-trimethylnaphthalene (THTMN: 177.77%), 2,4-di-tert-butylphenol (DTBP: 25%), 4-oxoisofolkone (OIFK: 116.66%,) 1,9-heptadecadiene-4,6-diyn-3-ol (HDD: 116.67%), β-damastrone (BDS: 116.67), and megastigmatrienone A (MSTA: 116.67%), was also increased by ˃100% and the nicotine level was decreased by 25% after 36 h of fermentation (Table 1). It is evident from the results of the GC-MS analysis that the production of NAECs increased with the growth of microbial cells in the RTLC, revealing a positive correlation between the production of NAECs and microbial growth. This suggests that microbial cells release a variety of enzymes into the environment, facilitating the conversion of metabolites in the RTLC into NAECs.

For the evaluation of the sensory quality of the FRTLC, cigarettes were made from the FRTLCs obtained after 0 h, 8 h, 16 h, 24 h, and 36 h. It was found that the cigarettes made from the FRTLCs fermented for up to 24 h received the highest sensory scores (37) compared to those made from the other FRTLCs (Supplementary Table S3). A score higher than 5 scale indicates better quality cigarettes produced from the FRTLC compared to the blank.

This indicates that *Klebsiella variicola* H8 and other microbial strains played vital roles in enhancing the aroma of the FRTLC.

**Figure 7:**
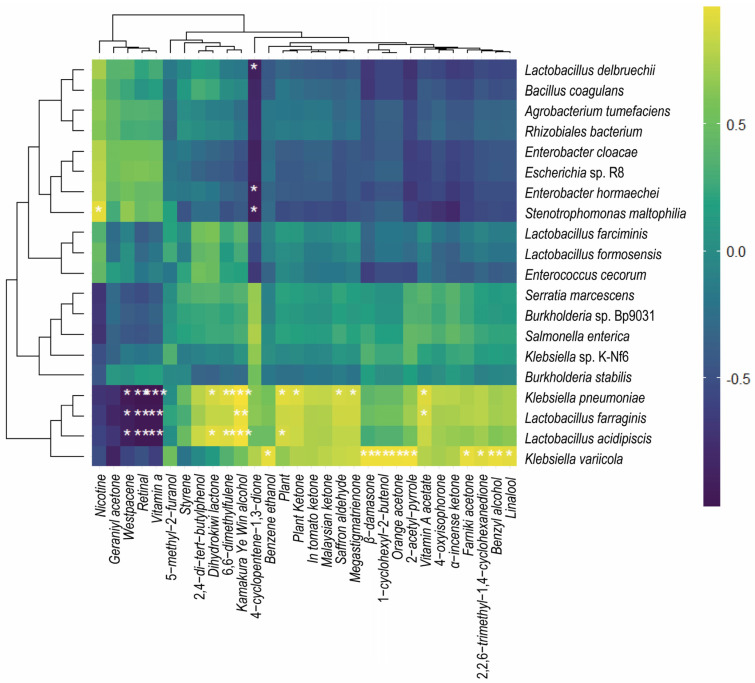
Heatmap presenting the correlation between the production of neutral aroma components and the growth of bacterial strains in the tobacco concentrate. The inoculation of *Klebsiella variicola* into the unsterilized RTLC enhanced the production of NAECs and dominated the microbial community of FRTLC. This dominance indirectly indicates its superiority over *Bacillus* in NAEC production. The asterisks (*) and (**) indicate significant and highly significant correlations, respectively. Negative and positive numbers indicate negative and positive correlations, respectively.

**Figure 8:**
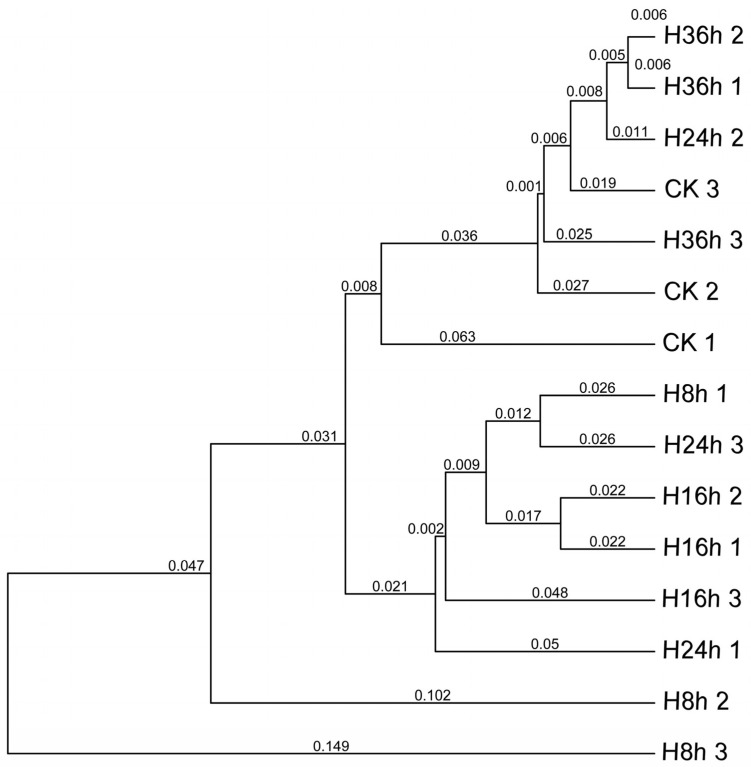
UPGMA cluster analysis of metabolic pathways.

The R version 4.3.2 was used to calculate the correlation between the production of NAECs and the bacterial strains in the FRTLC (Figure 7). The *Klebsiella variicola* H8 strain displayed a positive correlation with the production of most of the NAECs (Figure 7). UPGMA is often used in genetics to analyze DNA or cluster samples based on phenotypic traits and represents the evolutionary relationships among them through a dendrogram. The correlation analysis conducted using R software, cluster analysis of metabolic pathways using UPGMA (Figure 8), and the relative abundance of the dominant strains (Figure 2) showed a clear positive correlation between the production of NAECs and bacterial strains, as well as the metabolic pathways responsible for the production of NAECs and nicotine degradation.

The analysis of gene functional distances throughout the fermentation process provides valuable insights into the dynamic changes in the microbial community in the FRTLC and the production of NAECs. The gene functional distances at the initial (0 h) and final stages (after 36 h) of fermentation were similar, highlighting a substantial similarity in gene functions during these phases. Furthermore, after 8, 16, and 24 h of fermentation, the gene functional distances were closely clustered, implying similar functional attributes during these periods (Figure 8). These findings suggest that high-quality NAECs were produced in the FRTLC until the 24th hour of fermentation (Figure 8). The accumulation of NAECs decreased as the fermentation process progressed, reaching its lowest levels after 36 h of fermentation. This decline may be attributed to the depletion of nutrients in the FRTLC and the utilization of NAECs by the microbial community for their survival. The convergence in gene functional distances and the superior quality of the FRTLC after 24 h of fermentation collectively indicate that this duration produces the most favorable outcomes. The results of the correlation analysis using R software not only endorse this conclusion, but also clearly demonstrate that a strong correlation exists between the growth of microorganisms and the NAECs produced in the FRTLC solution (Figure 7).

The heatmap generated from the correlation analysis reveals that the *Klebsiella variicola* H8 strain exhibited significant positive correlations with the production of NAECs in the FRTLC (Figure 7) and increased their production levels (Table 1). This indicates that the enhanced growth of *Klebsiella variicola* H8 in the FRTLC is positively associated with an increased production of NAECs.

Similarly, other bacterial strains, including *Klebsiella pneumoniae*, also exhibited positive correlations with the production of different NAECs, such as Kamakura Ye Qin alcohol, 6-diyn-3-ol, DTBP, plant alcohol, phytone, saffron aldehyde, solanone, α-cyperone, phenylethanol, and linalool (Figure 7) and enhanced their production (Table 1). On the other hand, *Lactobacillus acidiphis*, *Lactobacillus farraginis*, and *Klebsiella pneumoniae* showed positive correlations with the production of NAECs, except for Westpac ene, with which they exhibited a significant negative correlation (Figure 7 and Table 1).

**Figure 9:**
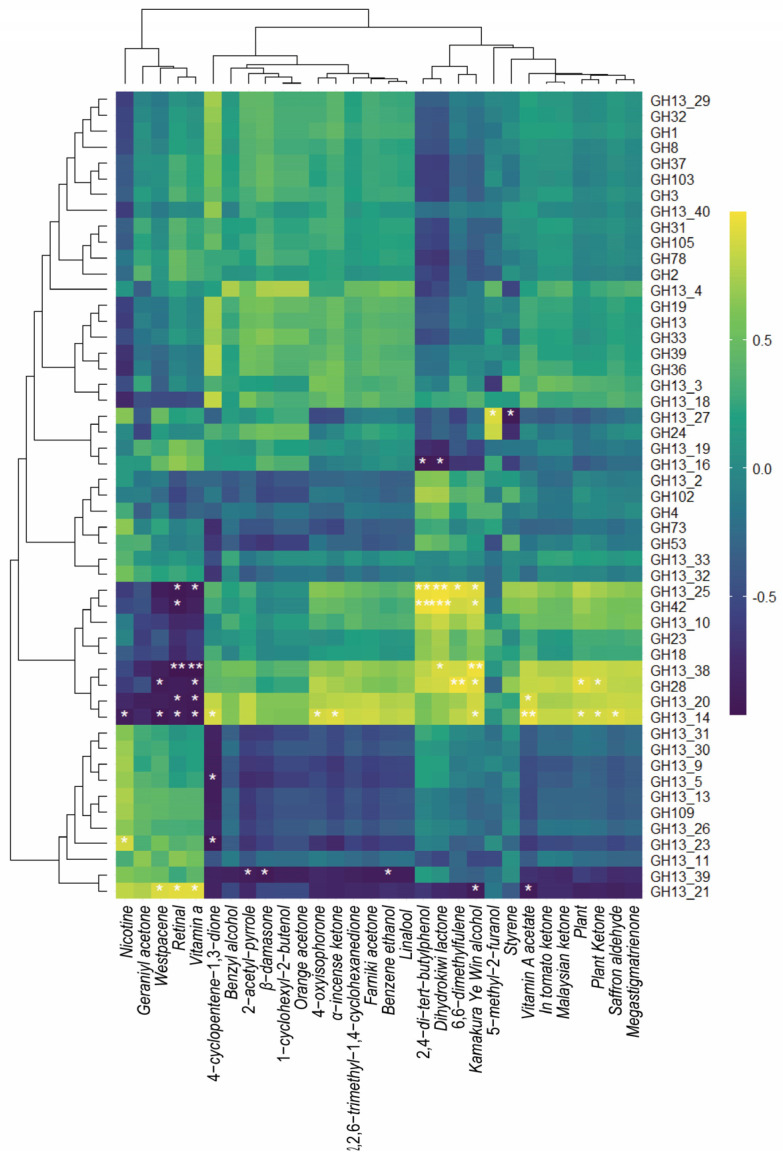
Heatmap depicting the correlation between GH enzymes and the production of neutral aroma compounds in the tobacco concentrate. The asterisks (*) and (**) indicate significant and highly significant correlations, respectively. Negative and positive numbers indicate negative and positive correlations, respectively.

The R software was also used to conduct a correlation analysis between GHs and the production of NAECs in the FRTLC (Figure 9). The heatmap of the correlation analysis reveals significant positive correlations between an increase in the production of MST, DHKL (192.86%), and DTBP (125%) with GH13-14, GH13-20, GH13-38, and GH28 during the fermentation of the RTLC (Figure 9 and Table 1). Additionally, a clear positive correlation was observed between NAECs and GH13-25, GH13-10, and GH42. Changes in the content of benzyl alcohol (58.33%), phenylethanol (47.36%), farnesyl acetone (45.98%), and solanone (47.25%) were positively correlated with GH13-14 and GH13-20, while changes in the westpac ene content were significantly negatively correlated with GH13-14, GH13-20, GH13-38, and GH28 (Figure 9, Table 1).

The GH13-14, GH13-20, GH13-38, GH13-25, GH13-10, GH42, and GH28 genes were identified as the main GH families expressed by *Klebsiella variicola* H8 during the fermentation of the RTLC and played a key role in the production of NAECs. Furthermore, the CAZy gene cluster annotation results demonstrated that the chromosome of the *Klebsiella variicola* H8 strain contains 24 genes related to the GH family, with 75 genes encoding multi-functional GHs (Supplementary Figure S2). In addition to these enzymes, various other enzymes such as CEs, GTs, and PLs were associated with the enhanced production of NAECs (Supplementary Table S2) and were annotated from the chromosome of *Klebsiella variicola* H8 (Supplementary Figure S2). Arylesterase (CE10: EC 3.1.1.3), acetyl xylan esterase (CE1: EC 3.1.1.72), and acetyl xylan esterase (CE4: EC 3.1.1.72) degrade cellulose and their genes have been annotated from the plasmid of *Klebsiella variicola* H8 (Supplementary Figure S3). 

In summary, the findings of this study support our hypothesis that the production of NAECs and changes in the chemical composition of FRTLCs result from the collaborative action of *Klebsiella variicola* H8 and other microorganisms, with *Klebsiella variicola* H8 playing a leading role.

## 4. Discussion

Microorganisms are known for their unique capabilities, which allow them to play diverse roles under varying growth conditions. This trait has been leveraged by the tobacco industry and other stakeholders to modify fundamental components, NAECs, and harmful chemicals such as nicotine in tobacco concentrates [40,41,42]. 

Tobacco-derived microorganisms can indeed modify the chemical composition of RTLCs and affect the sensory quality of the tobacco [43,44,45]. In this study, we investigated the correlation between the production of aroma compounds and changes in the microbial composition, and identified metabolic pathways and enzymes responsible for the production of these compounds in an FRTLC through a metagenomic analysis. *Klebsiella variicola* H8 was the major component of the microbial community employed for the fermentation of the RTLC and the optimization of fermentation time for the production of these NAECs.

The water-soluble total sugar content of the tobacco concentrate decreased rapidly and by approximately 19.106% between 8 and 16 h of fermentation. Additionally, the total alkaloid content initially decreased by approximately 5.22% after 16 h of fermentation, followed by an increase of about 4.72% after 36 h of fermentation (Supplementary Table S1). Nicotine, the primary alkaloid in tobacco, is mainly present as organic salts like malic and citric acid, with minimal free form. The initial increase and subsequent decrease in total alkaloids resulted from microorganisms initially degrading bound organic acid salts to release nicotine, which degraded only when these salts were used up [46]. Conversely, the production of NAECs reached its highest level after 24 h of fermentation (Table 1).

An analysis of the metagenomic sequencing data revealed the involvement of microbial metabolic pathways, such as the amino acid, lipid, carbohydrate, and terpene metabolism pathways, in the metabolism of macromolecules in the FRTLC. The changes in the abundance of the microbial community support these findings, indicating that the microbial organisms utilize the macromolecules present in the RTLC for growth, while also producing NAECs. For instance, carbohydrates in the FRTLC, represented by water-soluble sugar and reducing sugar, serve as crucial precursors for the production of NAECs [47]. The amino acids in the RTLC are significant contributors to the production of NAECs, generating substances like pyrrole, pyrazine, and furan, which have a substantial impact on the tobacco aroma [48]. Studies have reported that NAECs, such as megastigmatrienone, solanone, westpac ene, and dihydrokilli lactone (a carotenoid degradation product), play an essential role in the sensory quality of RTLCs [49,50].

The metagenomic data highlight that bacterial members of the microbial community in the RTLC possess numerous genes related to GHs, which enable them to hydrolyze the various carbohydrate substrates. For example, β-glucosidase hydrolyzes monoterpene glycosides and produces the corresponding monoterpene alcohols. Monoterpene alcohols are aromatic compounds that contribute to the aroma of various plants, including tobacco [51]. GHs are a class of enzymes that catalyze the hydrolysis of the glycosidic bonds in carbohydrates and produce smaller sugar units and oligosaccharides. These products are then utilized by microorganisms for the production of NAECs [52,53,54,55]. GTs are enzymes that catalyze the transfer of a sugar moiety, such as a glucose group, from a donor substrate to an acceptor molecule [56]. While GTs themselves may not directly produce aroma-enhancing compounds, they modify molecules by transferring a sugar moiety, and then these modified compounds serve as precursors for the production of aroma compounds during microbial fermentation [57,58,59]. In summary, an environment rich in GHs and GTs provides a foundation for the production of neutral aroma compounds through the hydrolysis of complex carbohydrates and the transfer of sugar moieties to appropriate molecules, which then act as precursors for the production of NAECs [60,61,62]. Our results also demonstrate that the production of NAECs is positively correlated with the abundance of GHs and GTs. Specifically, GH13-14, GH13-20, GH13-38, GH13-25, GH13-10, GH42, and GH28 emerged as the principal GH families involved in the aroma enhancement of the RTLC in this study and these enzymes have been annotated from the chromosome of the *Klebsiella variicola* H8 strain. It is noteworthy that the abundance of microbial organisms started decreasing after 16 h of fermentation along with a simultaneous decrease in the abundance of the aforementioned glycosidase genes. This subsequently resulted in a slowing in the accumulation of NAECs in the FRTLC, indicating that after consuming the macromolecules, microbial organisms start using the NAECs for their survival. 

In summary, the use of *Klebsiella variicola* H8 as a principal component of the microbial community strongly contributed to the production of NAECs in the FRTLC. Therefore, their combined efforts with other microbial organisms resulted in a higher production of NAECs in the FRTLC.

## 5. Conclusions

Metagenomic sequencing unveiled *Klebsiella variicola* as the dominant microbial species in the FRTLC solution, along with *Lactobacillus*, *Bacillus*, *Escherichia coli*, *Salmonella*, and *Serratia*. Through the metagenomic sequencing analysis, the most abundant genes in the microbial community of the tobacco concentrate were GH and GT genes at 31,724 and 29,819, respectively. Among them, the GH13-14, GH13-20, GH13-38, GH13-25, GH13-10, GH42, and GH28 genes were annotated from the chromosome of *Klebsiella variicola*, indicating the prominent role of this bacterium in the production of NAECs in the FRTLC.

In summary, metagenomic sequencing uncovered the dominance of *Klebsiella variicola* over other microbial organisms in terms of abundance and the production of NAECs. 

## Figures and Tables

**Table 1:** Neutral aroma components and flavor compounds of tobacco concentrates.

Number	CAS No.	Compound Name	Concentration (mg/mL)					Increase (%)
			0 h	8 h	16 h	24 h	36 h	
1	100-42-5	Styrene	0.28 ± 0.014	0.37 ± 0.015	0.40 ± 0.018	0.32 ± 0.016	0.47 ± 0.017	67.85
2	3857-25-8	5-Methyl-2-furanol	0.03 ± 0.002	0.02 ± 0.001	0.02 ± 0.002	0.03 ± 0.001	0.02 ± 0.002	NA
3	100-52-7	Benzaldehyde	0.03 ± 0.003	0.05 ± 0.002	0.04 ± 0.002	0.04 ± 0.001	0.04 ± 0.002	66.66
4	100-51-6	Benzyl alcohol	0.24 ± 0.016	0.27 ± 0.018	0.27 ± 0.021	0.38 ± 0.023	0.32 ± 0.001	58.33
5	122-78-1	Phenylacetaldehyde	0.67 ± 0.031	0.9 ± 0.044	0.84 ± 0.058	1.07 ± 0.062	0.98 ± 0.064	59.70
6	1072-83-9	2-Acetylpyrrole	0.17 ± 0.015	0.16 ± 0.014	0.20 ± 0.02	0.31 ± 0.019	0.21 ± 0.021	82.35
7	78-70-6	Linalool	0.08 ± 0.002	0.09 ± 0.003	0.09 ± 0.002	0.12 ± 0.019	0.10 ± 0.001	50
8	60-12-8	Phenylethanol	0.19 ± 0.008	0.21 ± 0.011	0.19 ± 0.009	0.28 ± 0.017	0.21 ± 0.016	47.36
9	1125-21-9	4-oxoisofolkone	0.06 ± 0.003	0.09 ± 0.004	0.10 ± 0.011	0.13 ± 0.015	0.12 ± 0.009	116.66
10	432-25-7	β-Cyclocitral	0.22 ± 0.012	0.27 ± 0.014	0.26 ± 0.008	0.36 ± 0.037	0.35 ± 0.025	63.63
11	56797-40-1	(Z)-7-Cetylenal	0.07 ± 0.004	0.09 ± 0.008	0.09 ± 0.011	0.12 ± 0.013	0.12 ± 0.009	71.42
12	116-26-7	Crocin aldehyde	0.08 ± 0.002	0.09 ± 0.004	0.09 ± 0.004	0.12 ± 0.009	0.11 ± 0.007	50
13	475-03-6	1,2,3,4-tetrahydro-1,1,6-trimethylnaphthalene	0.09 ± 0.004	0.09 ± 0.005	0.08 ± 0.008	0.25 ± 0.021	0.23 ± 0.022	177.77
14	54-11-5	Nicotine	0.08 ± 0.005	0.08 ± 0.003	0.06 ± 0.004	0.06 ± 0.004	0.06 ± 0.005	25
15	21852-80-2	1,9-Heptadecadiene-4,6-diyn-3-ol	0.06 ± 0.004	0.06 ± 0.004	0.06 ± 0.003	0.13 ± 0.009	0.16 ± 0.014	116.67
16	23726-93-4	β-Damastrone	0.06 ± 0.003	0.06 ± 0.002	0.07 ± 0.005	0.13 ± 0.008	0.06 ± 0.002	116.67
17	1883-13-2	(±)-3-hydroxylauric acid	-	-	0.05 ± 0.001	-	-	NA
18	54868-48-3	Solanone	0.91 ± 0.056	1.11 ± 0.079	1.07 ± 0.047	1.34 ± 0.102	1.26 ± 0.084	47.25
19	35044-68-9	α-Damarone	0.63 ± 0.032	0.75 ± 0.049	0.72 ± 0.036	0.91 ± 0.051	0.84 ± 0.047	44.44
20	3796-70-1	Geranyl acetone	0.26 ± 0.021	0.32 ± 0.025	0.31 ± 0.024	0.17 ± 0.019	0.24 ± 0.023	D
21	3879-26-3	Neroli acetone	-	-	-	0.07 ± 0.004	-	NA
22	96-76-4	2,4-di-tert-butylphenol	0.04 ± 0.003	0.05 ± 0.004	0.05 ± 0.003	0.09 ± 0.005	0.52 ± 0.004	125
23	17092-92-1	Dihydrokiwi lactone	0.42 ± 0.044	0.34 ± 0.041	0.33 ± 0.03	1.23 ± 0.098	0.58 ± 0.054	192.86
24	13215-88-8	Megastigmatrienone a	0.18 ± 0.009	0.20 ± 0.011	0.17 ± 0.01	0.39 ± 0.042	0.28 ± 0.03	116.67
25	13215-88-8	Megastigmatrienone b	1.03 ± 0.057	1.03 ± 0.061	0.98 ± 0.044	1.24 ± 0.081	1.09 ± 0.093	20.39
26	13215-88-8	Megastigmatrienone c	0.82 ± 0.052	0.96 ± 0.074	0.91 ± 0.063	1.13 ± 0.089	0.99 ± 0.076	37.80
27	13215-88-8	Megastigmatrienone d	0.18 ± 0.008	0.19 ± 0.012	0.25 ± 0.026	0.34 ± 0.015	0.28 ± 0.024	88.89
28	473-08-5	α-Cyperone	0.28 ± 0.014	0.33 ± 0.021	0.37 ± 0.027	0.43 ± 0.032	0.40 ± 0.028	53.57
29	102608-53-7	Phytol	1.67 ± 0.075	1.99 ± 0.09	1.93 ± 0.074	2.47 ± 0.095	2.50 ± 0.112	49.70
30	502-69-2	Phytone	0.43 ± 0.025	0.50 ± 0.034	0.48 ± 0.03	0.64 ± 0.051	0.61 ± 0.058	48.837
31	65646-68-6	4-hydroxyphenyl retinamide	0.36 ± 0.035	0.38 ± 0.031	0.34 ± 0.029	0.94 ± 0.084	0.37 ± 0.026	161.11
32	25360-09-2	potassium,2,6-ditert-butylphenolate	0.05 ± 0.002	-	0.05 ± 0.003	0.08 ± 0.005	-	NA
33	1117-52-8	farnesyl acetone	0.87 ± 0.084	0.92 ± 0.075	0.97 ± 0.082	1.27 ± 0.1	1.06 ± 0.087	45.98
34	112-39-0	Methyl hexadecanoate	1.05 ± 0.095	1.13 ± 0.102	1.15 ± 0.098	1.52 ± 0.137	1.31 ± 0.124	44.76
35	68-26-8	Vitamin a	0.89 ± 0.074	0.9 ± 0.085	0.85 ± 0.073	0.55 ± 0.042	0.47 ± 0.037	D
36	1898-13-1	CEMBRENE (Westpac ene)	0.25 ± 0.018	0.24 ± 0.02	0.23 ± 0.019	0.21 ± 0.207	0.20 ± 0.013	D
37	84-74-2	dibutyl phthalate	0.29 ± 0.018	0.31 ± 0.026	0.42 ± 0.037	0.65 ± 0.051	0.54 ± 0.048	124.14

Note: “-”, not detected; D, decreased; NA, not applicable. Three parallel groups analyzed each group of samples. Megastigmatrienone a, Megastigmatrienone b, Megastigmatrienone c and Megastigmatrienone d (CAS:13215-88-8) have the same CAS number because initially they were discovered as distinct compounds but were later found to be the same compound.

## Data Availability

The datasets generated and/or analyzed during the current study are available in the Genome Sequence Archive (Genomics, Proteomics & Bioinformatics 2021) in the National Genomics Data Center (Nucleic Acids Res 2022), China National Center for Bioinformation/Beijing Institute of Genomics, Chinese Academy of Sciences (GSA:CRA010523; https://bigd.big.ac.cn/gsa/browse/CRA010523, accessed on 5 April 2023).

## Supplementary Materials

The following supporting information can be downloaded at: https://www.mdpi.com/article/10.3390/metabo14030158/s1, Figure S1: Species Venn diagram; Figure S2: Correlation diagram between conventional chemical components and microorganisms; Figure S3: The map of chromosome of Klebsiella variicola H8 strain; Figure S4: The map of plasmid of *Klebsiella variicola* H8 strain; Table S1: Mass fraction of conventional chemical components of concentrated solution; Table S2: CAZy gene cluster annotation results; Table S3: Sensory rating scales for different fermentation times.

## Abbreviations

dihydrokiwi lactone (DHKL), 1,2,3,4-tetrahydro-1,1,6-trimethylnaphthalene (THTMN), 2,4-di-tert-butylphenol (DTBP), 4-oxoisofolkone (OIFK), 1,9-heptadecadiene-4,6-diyn-3-ol (HDD), β-damastrone (BDS), megastigmatrienone a (MST)

## References

[1] Lu J.J., Xue A.Q., Cao Z.Y., Yang S.J., Hu X.F. (2014). Diversity of Plant Growth-Promoting *Paenibacillus mucilaginosus* Isolated from Vegetable Fields in Zhejiang, China. Ann. Microbiol..

[2] Li H., Qu Y., Cao K., Xiong J., Hua K., Liu Z., Wang H., Yue L., Tang Y., Ding G. (2021). Cigarette Flavouring Regulation by Using Aroma-Producing Microorganism Isolated from Maotai Daqu. E3S Web Conf..

[3] Huang S., Wang M., Mao D.B., Rasool A., Jia C., Yang P., Han L., Yan M. (2022). Isolation, Identification and Characterization of Growth Parameters of *Pseudomonas putida* HSM-C2 with Coumarin-Degrading Bacteria. Molecules.

[4] Potts R.J., Bombick B.R., Meckley D.R., Ayres P.H., Pence D.H. (2010). A Summary of Toxicological and Chemical Data Relevant to the Evaluation of Cast Sheet Tobacco. Exp. Toxicol. Pathol..

[5] Wang W.S., Wang Y., Yang L.J., Liu B., Lan M., Sun W. (2005). Studies on Thermal Behavior of Reconstituted Tobacco Sheet. Thermochim. Acta.

[6] Brown C.J., Cheng J.M. (2014). Electronic Cigarettes: Product Characterization and Design Considerations. Tob. Control.

[7] Zeng T., Liu Y.X., Jiang Y.F., Zhang L., Zhang Y., Zhao L., Jiang X., Zhang Q. (2023). Advanced Materials Design for Adsorption of Toxic Substances in Cigarette Smoke. Adv. Sci..

[8] Shu M.H., Fan H., Liu J.L., Yang Y., Xu S., Gao Y., Zhou G. (2016). High-Throughput Bacterial Analysis on Aqueous Extract of Waste Tobacco Scrap. Tob. Sci. Technol..

[9] Liu H.G., He H.L., Cheng C.H., Liu J., Shu M., Jiao Y., Tao F., Zhong W. (2015). Diversity Analysis of the Bacterial Community in Tobacco Waste Extract during Reconstituted Tobacco Process. Appl. Microbiol. Biotechnol..

[10] Ruan A., Min H. (2005). Studies on Microbiological Degradation of Tobacco Tar. J. Environ. Sci. Health Part A Toxic/Hazard. Subst. Environ. Eng..

[11] Zhang Z.L., Mei X.T., He Z.L., Xie X., Yang Y., Mei C., Xue D., Hu T., Shu M., Zhong W. (2022). Nicotine Metabolism Pathway in Bacteria: Mechanism, Modification, and Application. Appl. Microbiol. Biotechnol..

[12] Zheng T.F., Zhang Q.Y., Wu Q.Y., Li D., Wu X., Li P., Zhou Q., Cai W., Zhang J., Du G. (2022). Effects of Inoculation with Acinetobacter on Fermentation of Cigar Tobacco Leaves. Front. Microbiol..

[13] Gravely L.E., Geiss V.L. (1983). Microbial digestion of tobacco materials using mixed cultures.

[14] Huang Y.G., Wu Q., Xu Y. (2014). Isolation and Identification of a Black Aspergillus Strain and the Effect of Its Novel Protease on the Aroma of Moutai-Flavoured Liquor. J. Inst. Brew..

[15] Wang L. (2022). Research Trends in Jiang-Flavor Baijiu Fermentation: From Fermentation Microecology to Environmental Ecology. J. Food Sci..

[16] Li Z.X., Xu L.Y., Zhu Y.P., Li J., Li X. (2021). Optimization of Synthesis Technology of Galacto-Oligosaccharides by Heterologous Expression of β-Galactosidase from Klebsiella. J. Chin. Inst. Food Sci. Technol..

[17] Ansari S.A., Satar R. (2012). Recombinant β-Galactosidases—Past, Present and Future: A Mini Review. J. Mol. Catal. B Enzym..

[18] Lu L.L., Guo L.C., Wang K., Liu Y., Xiao M. (2020). β-Galactosidases: A Great Tool for Synthesizing Galactose-Containing Carbohydrates. Biotechnol. Adv..

[19] Oliveira C., Guimarães P.M.R., Domingues L. (2011). Recombinant Microbial Systems for Improved β-Galactosidase Production and Biotechnological Applications. Biotechnol. Adv..

[20] Wöhner G., Wöber G. (1978). Pullulanase, an Enzyme of Starch Catabolism, Is Associated with the Outer Membrane of Klebsiella. Arch. Microbiol..

[21] Boyd J., Turvey J.R. (1977). Isolation of a Poly-α-l-Guluronate Lyase from Klebsiella Aerogenes. Carbohydr. Res..

[22] Caffrey A., Ebeler S.E. (2021). The Occurrence of Glycosylated Aroma Precursors in *Vitis vinifera* Fruit and *Humulus lupulus* Hop Cones and Their Roles in Wine and Beer Volatile Aroma Production. Foods.

[23] Chen D.J. (2020). Characteristic Analysis of Carbapenem-Resistant *Klebsiella pneumoniae* and Study on the Mechanism of Tigecycline Resistance. Master’s Thesis.

[24] Zhong Y.M., Luo X.Y., Li Y.M., Li H.L., Jian Z.J., Yan Q., Liu W.E. (2023). Clinical characteristics of patients with *Klebsiella variicola* infection. Chin. J. Infect. Control.

[25] Huang H., Huang Y.L., Gu D.X., Zhang R., Zhou H.W. (2019). Analysis of drug resistance genes and molecular typing of carbapenem-resistant *Klebsiella variicola* strains. Chin. J. Microbiol. Immunol..

[26] Moreno F.L., Quintanilla-Carvajal M.X., Sotelo L.I., Osorio C., Raventós M., Hernández E., Ruiz Y. (2015). Volatile Compounds, Sensory Quality and Ice Morphology in Falling-Film and Block Freeze Concentration of Coffee Extract. J. Food Eng..

[27] Fan X., Zi W.H., Ao J., Li B., Qiao J., Wang Y., Nong Y. (2022). Analysis and Application Evaluation of the Flavour-Precursor and Volatile-Aroma-Component Differences between Waste Tobacco Stems. Heliyon.

[28] Zi W.H., Zhang X.L., Peng J.H., Zhang L., Long M., Zuo J. (2013). Optimization of Microwave Drying Biomass Material of Stem Granules from Waste Tobacco Using Response Surface Methodology. Dry Technol..

[29] Pang X.N., Han B.Z., Huang X.N., Zhang X., Hou L.F., Cao M., Gao L.J., Hu G.H., Chen J.Y. (2018). Effect of the Environment Microbiota on the Flavour of Light-Flavour Baijiu during Spontaneous Fermentation. Sci. Rep..

[30] Su C., Zhang K.Z., Cao X.Z., Yang J.G. (2020). Effects of *Saccharomycopsis fibuligera* and *Saccharomyces cerevisiae* Inoculation on Small Fermentation Starters in Sichuan-Style Xiaoqu Liquor. Food Res. Int..

[31] Neuman H., Debelius J.W., Knight R., Koren O. (2015). Microbial Endocrinology: The Interplay between the Microbiota and the Endocrine System. FEMS Microbiol. Rev..

[32] Williams C.L., Garcia-Reyero N., Martyniuk C.J., Tubbs C.W., Bisesi J.H. (2020). Regulation of Endocrine Systems by the Microbiome: Perspectives from Comparative Animal Models. Gen. Comp. Endocrinol..

[33] Liu A.M., Yuan K.L., Li Q., Liu S., Li Y., Tao M., Xu H., Tian J., Guan S., Zhu W. (2022). Metabolomics and Proteomics Revealed the Synthesis Difference of Aroma Precursors in Tobacco Leaves at Various Growth Stages. Plant Physiol. Biochem..

[34] Fan J.H., Kong G.H., Yao H., Wu Y., Zhao G., Li F., Zhang G. (2023). Widely Targeted Metabolomic Analysis Reveals That Volatile Metabolites in Cigar Tobacco Leaves Dynamically Change during Fermentation. Biochem. Biophys. Rep..

[35] Wen C., Hu W.R., Li P.H., Liu J., Zhang Q., Zhou Q., Luo C., Li D. (2022). Effects of Fermentation Medium on Cigar Filler. Front. Bioeng. Biotechnol..

[36] Wu X., Hu Y.Q., Wang Q., Liu J., Fang S., Huang D., Pang X., Cao J., Gao Y., Ning Y. (2023). Study on the Correlation between the Dominant Microflora and the Main Flavor Substances in the Fermentation Process of Cigar Tobacco Leaves. Front. Microbiol..

[37] Chen J., He X., Zhang X.Y., Chen Y., Zhao L., Su J., Qu S., Ji X., Wang T., Li Z. (2021). The Applicability of Different Tobacco Types to Heated Tobacco Products. Ind. Crops Prod..

[38] Tang Z.X., Chen L.L., Chen Z.B., Fu Y., Sun X., Wang B., Xia T. (2020). Climatic Factors Determine the Yield and Quality of Honghe Flue-Cured Tobacco. Sci. Rep..

[39] Kurt D. (2021). Impacts of Environmental Variations on Quality and Chemical Contents of Oriental Tobacco. Beiträge zur Tab. Int./Con. to Tob. Res..

[40] Li J.J., Zhao Y.Y., Qin Y.Q., Shi H. (2020). Influence of Microbiota and Metabolites on the Quality of Tobacco during Fermentation. BMC Microbiol..

[41] Huang W.J., Yang J.K., Duan Y.Q., Gu W., Gong X., Zhe W., Su C., Zhang K.Q. (2010). Bacterial Diversities on Unaged and Aging Flue-Cured Tobacco Leaves Estimated by 16S RRNA Sequence Analysis. Appl. Microbiol. Biotechnol..

[42] Gong X.W., Yang J.K., Duan Y.Q., Dong J.Y., Zhe W., Wang L., Li Q.H., Zhang K.Q. (2009). Isolation and Characterization of *Rhodococcus* sp. Y22 and Its Potential Application to Tobacco Processing. Res. Microbiol..

[43] Zhang Q.Y., Zheng T.F., Yang Z., Yang S., Cai W., Li P., Huang Y., Zhang J., Li D. (2023). Analysis of the Structure and Metabolic Function of Microbial Community in Cigar Tobacco Leaves in Agricultural Processing Stage. Front. Microbiol..

[44] Liu F., Wu Z.Y., Zhang X.P., Xi G., Zhao Z., Lai M., Zhao M. (2021). Microbial Community and Metabolic Function Analysis of Cigar Tobacco Leaves during Fermentation. Microbiologyopen.

[45] Jia Y., Liu Y.F., Hu W.R., Cai W., Zheng Z., Luo C., Li D. (2023). Development of Candida Autochthonous Starter for Cigar Fermentation via Dissecting the Microbiome. Front. Microbiol..

[46] Huang X.L., Lei L.P., Xai Z.Y., Wu Y.P., Yang K.J. (2010). Preliminary study on the mechanism of degradation of nicotine and increasing scent of *Arthrobacter* sp.. Southwest Chin. J. Agric. Sci..

[47] Ardö Y. (2006). Flavour Formation by Amino Acid Catabolism. Biotechnol. Adv..

[48] Rodríguez-Bustamante E., Sánchez S. (2007). Microbial Production of C13-Norisoprenoids and Other Aroma Compounds via Carotenoid Cleavage. Crit. Rev. Microbiol..

[49] Johnson R.R., Nicholson J.A. (1965). The Structure, Chemistry, and Synthesis of Solanone. A New Anomalous Terpenoid Ketone from Tobacco. J. Org. Chem..

[50] Demole E., Berthet D. (1972). A Chemical Study of Burley Tobacco Flavour (*Nicotiana tabacum* L.). I. Volatile to Medium-volatile Constituents (b.p. ≦ 84°/0.001 Torr. Helv. Chim. Acta.

[51] Ahmed A., Aslam M., Ashraf M., ul-Hassan Nasim F., Batool K., Bibi A. (2017). Microbial β-Glucosidases: Screening, Characterization, Cloning and Applications. J. Appl. Environ. Microbiol..

[52] Wahlberg I., Karlsson K., Austin D.J., Junker N., Roeraade J., Enzell C.R., Johnson W.H. (1977). Effects of Flue-Curing and Ageing on the Volatile, Neutral and Acidic Constituents of Virginia Tobacco. Phytochemistry.

[53] Sathya T.A., Khan M. (2014). Diversity of Glycosyl Hydrolase Enzymes from Metagenome and Their Application in Food Industry. J. Food Sci..

[54] Abdul Manas N.H., Md (2018). Illias, R.; Mahadi, N.M. Strategy in Manipulating Transglycosylation Activity of Glycosyl Hydrolase for Oligosaccharide Production. Crit. Rev. Biotechnol..

[55] Amin K., Tranchimand S., Benvegnu T., Abdel-Razzak Z., Chamieh H. (2021). Glycoside Hydrolases and Glycosyltransferases from Hyperthermophilic Archaea: Insights on Their Characteristics and Applications in Biotechnology. Biomolecules.

[56] Sun W., Yang J., Zhou J., Xu D., Bai R., Zhang G., Ma Y., Shi H. (2015). Effect of Different Nitrogen Forms on Nitrate Nitrogen Content and TSNAs Formation in Tobacco. Acta Tabacaria Sin..

[57] Schwab W., Fischer T.C., Giri A., Wüst M. (2015). Potential Applications of Glucosyltransferases in Terpene Glucoside Production: Impacts on the Use of Aroma and Fragrance. Appl. Microbiol. Biotechnol..

[58] Liang Z.J., Fang Z.X., Pai A., Luo J., Gan R., Gao Y., Lu J., Zhang P. (2022). Glycosidically Bound Aroma Precursors in Fruits: A Comprehensive Review. Crit. Rev. Food Sci. Nutr..

[59] Zhu F.M., Du B., Ma Y.G., Li J. (2017). The Glycosidic Aroma Precursors in Wine: Occurrence, Characterization and Potential Biological Applications. Phytochem. Rev..

[60] Zhang Q., Luo C., Li D., Cai W. (2020). Research Progress in Curing and Fermentation Technology for Cigar Tobacco Leaf Production. Acta Tabacaria Sin..

[61] Yang Y., Peng Q.R., Ou M.Y., Wu Y., Fang J. (2018). Research Progress in Tobacco Fermentation. J. Biosci. Med..

[62] Xia Y.N., Shuang Q. (2022). Metagenomic Analysis of Microbial Diversity and Key Flavor-Related Genes in Solid-State Fermented Jujube Mash for Jujube Wine. Shipin Kexue/Food Sci..

