# Supplementary material for: Unravel the Supremacy of Klebsiella variicola over Native Microbial Strains for Aroma-Enhancing Compound Production in Reconstituted Tobacco Concentrate through Metagenomic Analysis
Source: Metabolites. 2024 Mar 8;14(3):158. doi: 10.3390/metabo14030158 (PMC10971923; doi:10.3390/metabo14030158)
Supplement: Supplementary file 1 [file metabolites-14-00158-s001.zip › metabolites-2813845-supplementary.pdf]

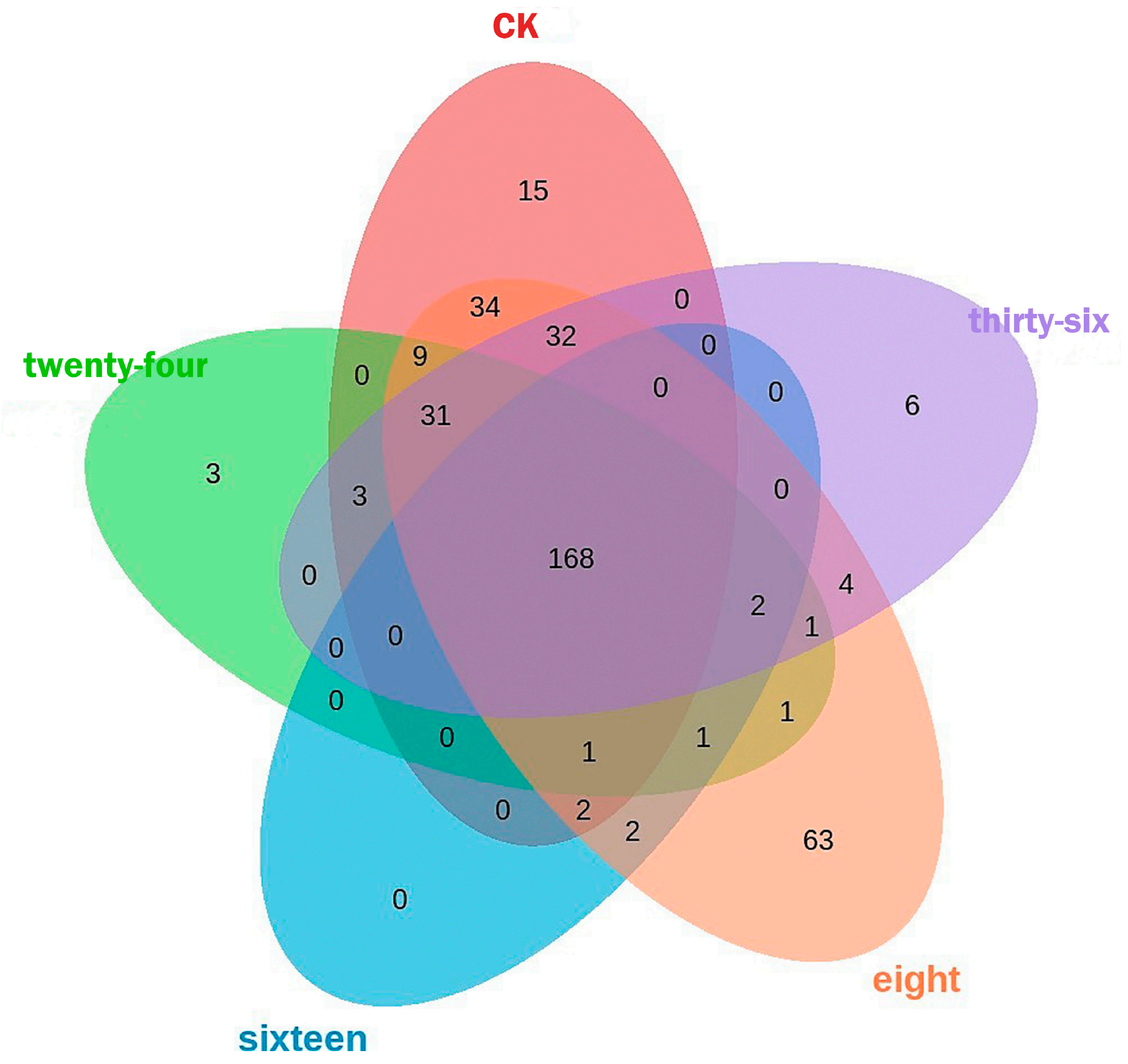

Supplementary Figure S1. Species Venn diagram.

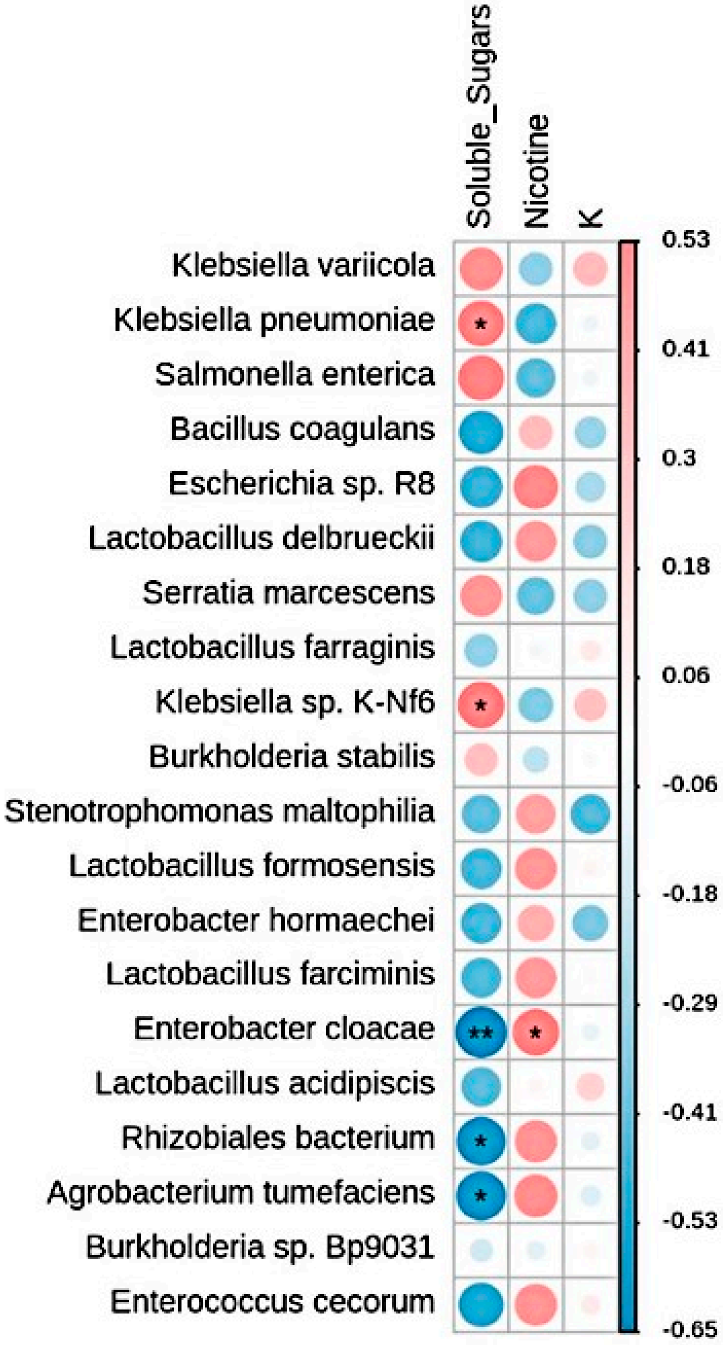

Supplementary Figure S2. Correlation diagram between conventional chemical components and microorganisms. (The red in the figure represents a positive correlation, while the blue represents a negative correlation. The darker the color, the higher the correlation). The asterisks (*) and (**) indicate significant and highly significant correlations, respectively.

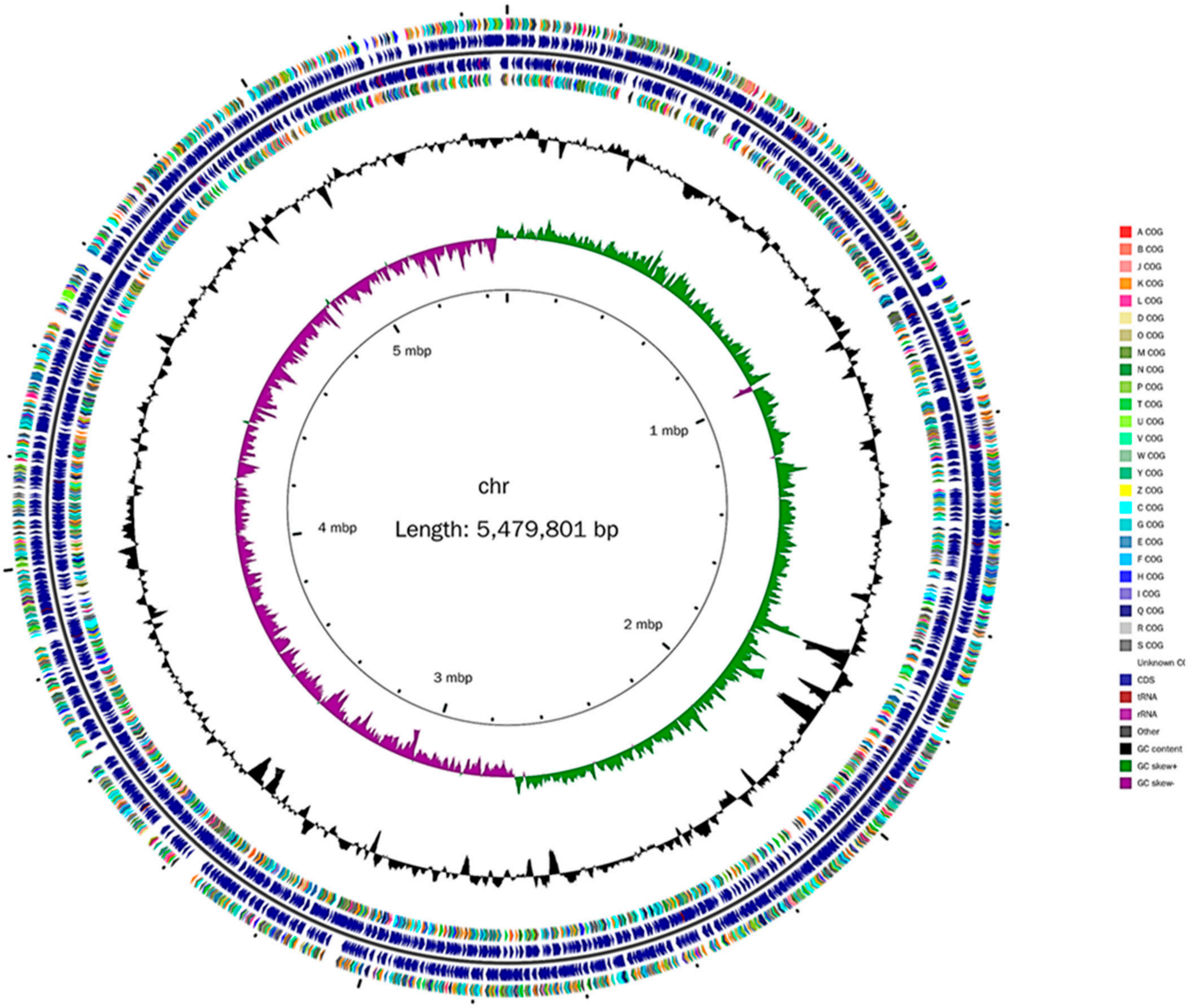

Supplementary Figure S3. The map of chromosome of *Klebsiella variicola* H8 strain.

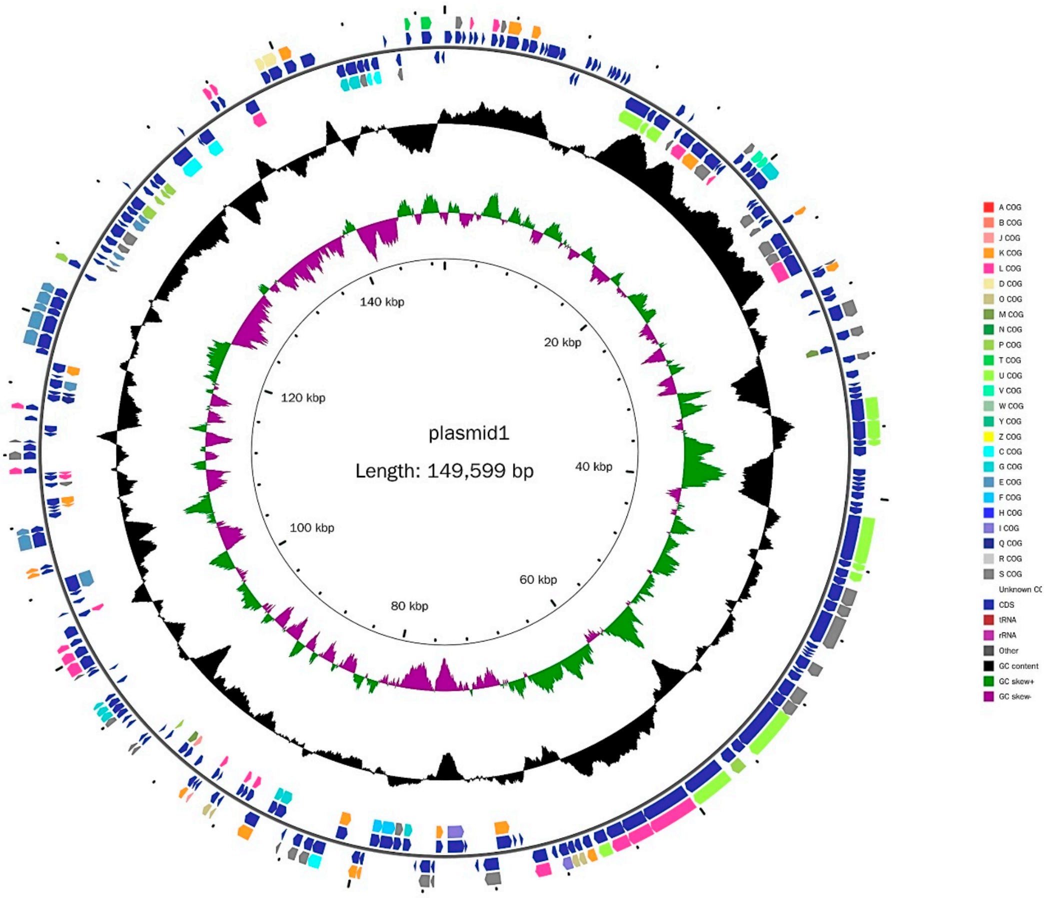

Supplementary Figure S4. The map of plasmid of *Klebsiella variicola* H8 strain.

Supplementary Tables

Supplementary Table S1. Mass fraction of conventional chemical components of concentrated solution

Sample	Water-soluble Total Sugar （%）	Total Alkaloids （%）	Potassium （%）
0 h	6.83	1.34	3.63
8 h	6.49	1.30	3.66
16 h	5.25	1.27	3.64
24 h	4.78	1.32	3.67

36 h	4.37	1.33	3.80

Supplementary Table S2. CAZy gene cluster annotation results

Type	Family	Gene cluster	Associated protein
chr	CE	CE1	acetyl xylan esterase (EC 3.1.1.72)
		CE10	arylesterase (EC 3.1.1.-)
		CE11	UDP-3-0-acyl N-acetylglucosamine deacetylase (EC 3.5.1.-).
		CE14	N-acetyl-1-D-myo-inosityl-2-amino-2-deoxy-alpHa-D- glucopyranoside deacetylase (EC 3.5.1.89)
		CE3	acetyl xylan esterase (EC 3.1.1.72).
		CE7	acetyl xylan esterase (EC 3.1.1.72)
		CE8	pectin methylesterase (EC 3.1.1.11).
		CE9	N-acetylglucosamine 6-pHospHate deacetylase (EC 3.5.1.25)
	GH	GH1	beta-glucosidase (EC 3.2.1.21)
		GH102	peptidoglycan lytic transglycosylase (EC 3.2.1.-)
		GH103	peptidoglycan lytic transglycosylase (EC 3.2.1.-)
		GH105	unsaturated rhamnogalacturonyl hydrolase (EC 3.2.1.-)
		GH109	alpHa-N-acetylgalactosaminidase (EC 3.2.1.49)
		GH13	alpHa-amylase (EC 3.2.1.1)
		GH18	chitinase (EC 3.2.1.14)
		GH19	chitinase (EC 3.2.1.14).
		GH2	beta-galactosidase (EC 3.2.1.23)
		GH23	lysozyme type G (EC 3.2.1.17)
		GH24	lysozyme (EC 3.2.1.17)
		GH28	polygalacturonase (EC 3.2.1.15)
		GH3	beta-glucosidase (EC 3.2.1.21)
		GH31	alpHa-glucosidase (EC 3.2.1.20)
		GH32	invertase (EC 3.2.1.26)
		GH33	sialidase or neuraminidase (EC 3.2.1.18)
		GH36	alpHa-galactosidase (EC 3.2.1.22)
		GH37	alpHa,alpHa-trehalase (EC 3.2.1.28).
		GH39	alpHa-L-iduronidase (EC 3.2.1.76)
		GH4	maltose-6-pHospHate glucosidase (EC 3.2.1.122)
		GH42	beta-galactosidase (EC 3.2.1.23)
		GH53	endo-beta-1,4-galactanase (EC 3.2.1.89).
		GH73	peptidoglycan hydrolase with endo-beta-N-acetylglucosaminidase specificity (EC 3.2.1.-)
		GH77	amylomaltase or 4-alpHa-glucanotransferase (EC

			2.4.1.25)
		GH78	alpHa-L-rhamnosidase (EC 3.2.1.40)
		GH8	chitosanase (EC 3.2.1.132)
chr		GT19	lipid-A-disaccharide synthase (EC 2.4.1.182).
		GT2	cellulose synthase (EC 2.4.1.12)
		GT20	alpHa,alpHa-trehalose-pHospHate synthase [UDP-forming] (EC 2.4.1.15)
		GT26	UDP-ManNAcA: beta-N-acetyl mannosaminuronyltransferase (EC 2.4.1.-)
		GT28	1,2-diacylglycerol 3-beta-galactosyltransferase (EC 2.4.1.46)
		GT30	CMP-beta-KDO: alpHa-3-deoxy-D-manno-octulosonic-acid (KDO) transferase (EC 2.4.99.-).
	GT	GT35	glycogen or starch pHospHorylase (EC 2.4.1.1).
		GT4	sucrose synthase (EC 2.4.1.13)
		GT5	UDP-Glc: glycogen glucosyltransferase (EC 2.4.1.11)
		GT51	murein polymerase (EC 2.4.1.129).
		GT56	TDP-Fuc4NAc: lipid II Fuc4NAc transferase (EC 2.4.1.-)
		GT73	CMP-beta-KDO: alpHa-3-deoxy-D-manno-octulosonic-acid (KDO) transferase (EC 2.4.99.-).
		GT83	undecaprenyl pHospHate-alpHa-L-Ara4N: 4-amino-4-deoxy-beta-L-arabinosyltransferase (EC 2.4.2.-)
		GT9	lipopolysaccharide N-acetylglucosaminyltransferase (EC 2.4.1.56)
	PL	PL22	oligogalacturonate lyase / oligogalacturonide lyase (EC 4.2.2.6)
		CE10	arylesterase (EC 3.1.1.-)
plasmid	CE	CE1	acetyl xylan esterase (EC 3.1.1.72)
		CE4	acetyl xylan esterase (EC 3.1.1.72)
	GH	GH23	lysozyme type G (EC 3.2.1.17)

Supplementary Table S3. Sensory rating scales for different fermentation times

Sample	Aroma quality	Aroma quantity	Smoke	woody odor	aftertaste	bad odor	irritation	Total score
0h	5.0	5.0	5.0	5.0	5.0	5.0	5.0	35.0
8h	5.0	5.5	5.0	5.0	5.0	5.0	5.0	35.5

16h	5.0	5.5	5.0	5.0	5.1	5.0	5.5	36.1
24h	5.5	5.5	5.4	5.3	5.3	4.5	5.0	37.0
36h	5.3	5.2	4.7	4.6	5.0	4.8	4.5	34.1
